# High definition spectral domain optical coherence tomography of retinal pigment epithelial rip in a case of sympathetic ophthalmia

**DOI:** 10.1186/1869-5760-3-19

**Published:** 2013-01-21

**Authors:** Padmamalini Mahendradas, Kavitha Avadhani, Bhanumathi Madhavarao, Anand Vinekar, Kodandaramaiah Sahana, Rohit Shetty, Bhujang K Shetty

**Affiliations:** 1 Department of Uveitis and Ocular Immunology, Narayana Nethralaya and Post Graduate Institute of Ophthalmology, 121/C, Chord Road, Rajaji Nagar 1st ‘R’ Block, 560010, Bangalore, India

**Keywords:** Sympathetic ophthalmia, RPE rip, SD-OCT

## Abstract

**Background:**

We are reporting a case of granulomatous panuveitis in the right eye following penetrating injury to the left eye.

**Findings:**

A 34-year-old female was diagnosed to have sympathetic ophthalmia on treatment with systemic steroids. Vision did not improve in spite of aggressive systemic steroid therapy. On examination, patient had large retinal pigment epithelial rip nasal to the disc with exudative retinal detachment which was documented with FFA, ICG, and OCT. RPE rip is responsible for the persistent exudative retinal detachment in the right eye.

**Conclusions:**

RPE rip can cause decreased vision due to persistence of retinal detachment in a case of sympathetic ophthalmia.

## Findings

### Introduction

Sympathetic ophthalmia (SO) is a bilateral granulomatous panuveitis in which the sympathising eye suffers granulomatous panuveitis after penetrating injury to the fellow eye [1]. We report a case of SO with retinal pigment epithelial (RPE) rip documented with high definition spectral domain optical coherence tomography (OCT). Informed consent was obtained from the patient for publication.

### Case report

Institutional ethics committee approval was obtained for the study of a 34-year-female diagnosed to have right eye SO since 1 month presented to us with insignificant improvement of vision in the right eye despite treatment with systemic steroids for 4 weeks. She stated a history of trauma to the left eye 3 months back and had undergone enucleation of the injured eye on the 17th day after the injury.

**Figure 1:**
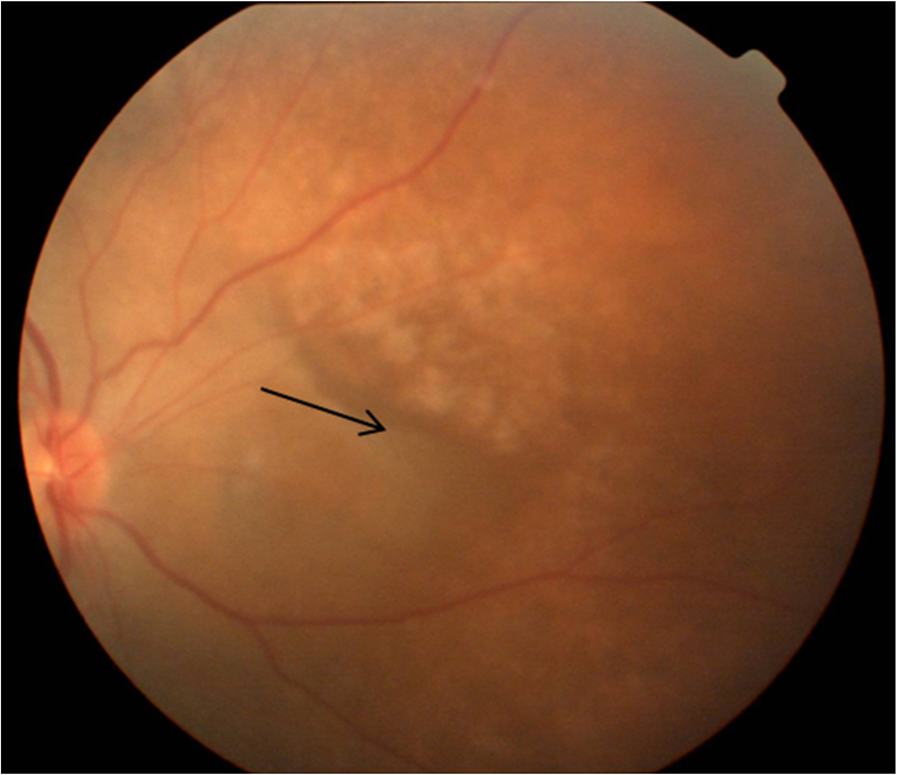
**Colour fundus photograph.** This shows an area of RPE rip nasal to the optic disc (shown in black arrow).

On examination, visual acuity of the right eye was counting fingers 0.5 metres. There was a prosthesis in the left eye socket. Intraocular pressure was 15 mmHg in the right eye. Slit lamp biomicroscopic examination revealed pigments on the anterior lens capsule with vitritis ++, haze ++, internal limiting membrane folds in the right eye with retinal pigment epithelial changes and serous elevation of the retina in the posterior pole with a large area of retinal pigment epithelial rip nasal to the disc (Figure 1).

**Figure 2:**
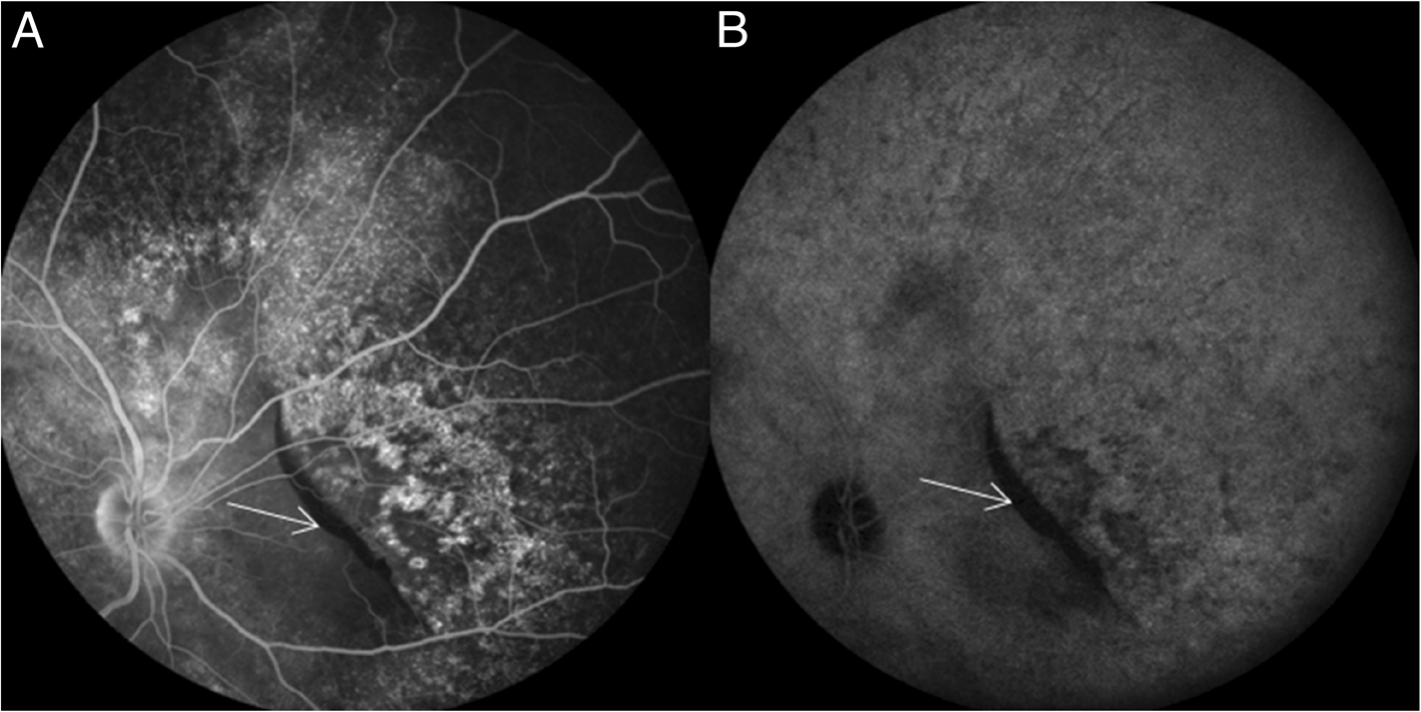
**Combined FFA and ICG photograph.** FFA (**A**) and ICG (**B**). This shows a blocked fluorescence corresponding to the area of RPE rip nasal to the disc (white arrow) in the right eye.

**Figure 3:**
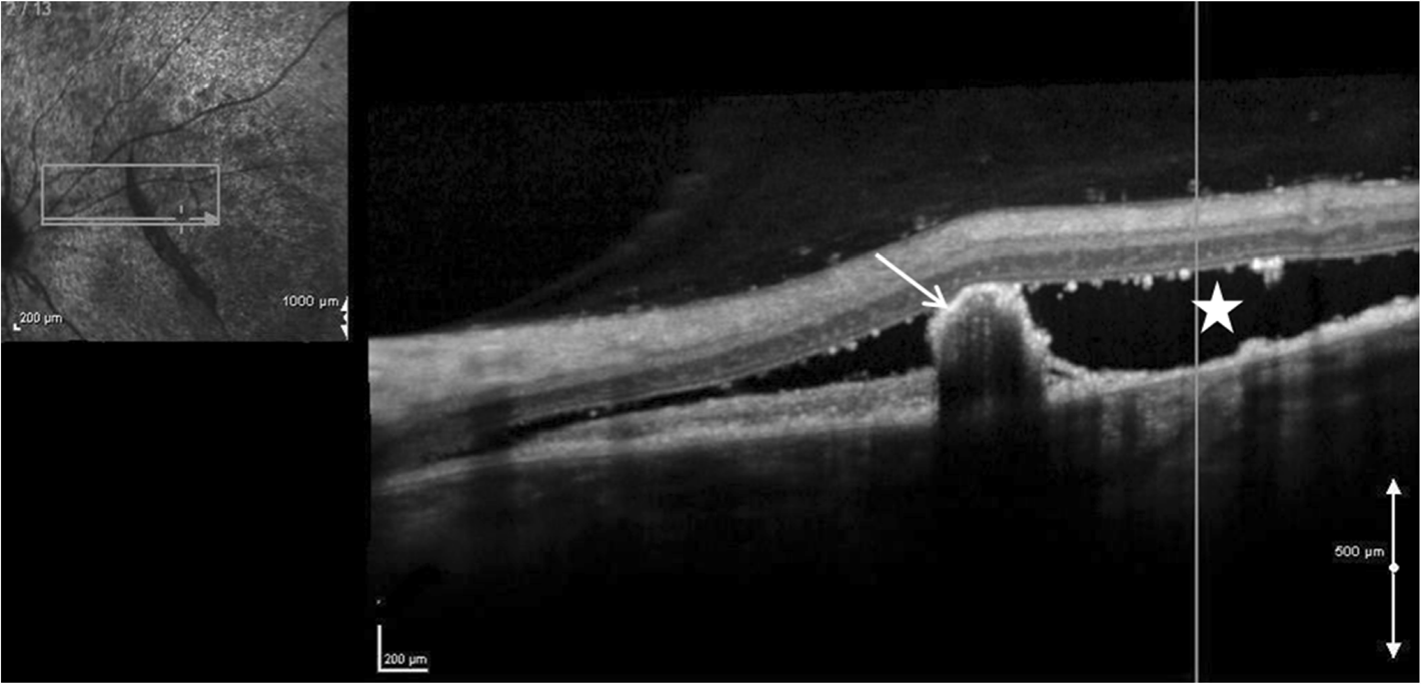
**High definition spectral domain optical coherence tomography.** This shows hyporeflective area suggestive of serous detachment (white star) and adjoining hyper-reflective area corresponding to RPE rip in the right eye (white arrow).

A combined fluorescein angiography and indocyanine green angiography revealed speckled transmission hyperfluorescence along with hypofluorescence corresponding to the rolled up edge of the rip in the early phase with more marked speckled transmission hyperfluorescence in the surroundings in the late phase with persistence of hypofluorescence corresponding to the rolled up edge of the rip in the late phase (Figure 2). OCT revealed hyper-reflective echoes in the vitreous, hyporeflective lesion suggestive of serous detachment with hyper-reflective area corresponding to RPE rip (Figure 3).

### Discussion

Reversible retinal changes such as serous retinal detachment and elongation of photoreceptors have been reported [1] in the acute stage of SO on spectral domain optical coherence tomography. Some other changes reported include disorganisation and thinning of the inner retina, and pronounced disintegration of the RPE and choriocapillaries (‘waterfall effect’) [2]. Peripheral rips have been reported to occur following uveal effusion syndrome [3], panuveitis, trauma and acute retinal necrosis [4]. Mechanical stretching of the RPE secondary to choroidal swelling has been suggested as the mechanism of rips due to many choroidal effusions [3]. Sympathetic ophthalmia is associated with granulomatous inflammation of the choroid with significant increase in choroidal thickness. We hypothesise that a similar stretching mechanism of the RPE had a role to play in the rip seen in our case. We also believe that the pigment epithelial rip may itself have caused some exudative detachment owing to a loss of the RPE barrier function [5] in addition to that caused by the primary disease, thus complicating both the clinical picture and the anatomic and visual recovery despite adequate treatment.

### Conclusion

RPE rip is an irreversible change in sympathetic ophthalmia in addition to acute reversible retinal changes. Medline search did not reveal the report of RPE rip in sympathetic ophthalmia earlier.

## Competing interests

The authors declare that they have no competing interest.

## Authors' contributions

PM designed the study, collected the data, and drafted the manuscript. KA collected the data and drafted the manuscript. BM collected the data and edited the manuscript. AV collected the data. KS, RS, and BS prepared the manuscript. All authors read and approved the final manuscript.
